# Asymmetries in visual acuity around the visual field

**DOI:** 10.1167/jov.21.1.2

**Published:** 2021-01-05

**Authors:** Antoine Barbot, Shutian Xue, Marisa Carrasco

**Affiliations:** 1 Department of Psychology, New York University, New York, NY, USA; 2 Center for Neural Science, New York University, New York, NY, USA; 3 Spinoza Centre for Neuroimaging, Amsterdam, Netherlands

**Keywords:** spatial frequency, performance fields, visual acuity, spatial vision, isoeccentricity

## Abstract

Human vision is heterogeneous around the visual field. At a fixed eccentricity, performance is better along the horizontal than the vertical meridian and along the lower than the upper vertical meridian. These asymmetric patterns, termed *performance fields*, have been found in numerous visual tasks, including those mediated by contrast sensitivity and spatial resolution. However, it is unknown whether spatial resolution asymmetries are confined to the cardinal meridians or whether and how far they extend into the upper and lower hemifields. Here, we measured visual acuity at isoeccentric peripheral locations (10 deg eccentricity), every 15° of polar angle. On each trial, observers judged the orientation (± 45°) of one of four equidistant, suprathreshold grating stimuli varying in spatial frequency (SF). On each block, we measured performance as a function of stimulus SF at 4 of 24 isoeccentric locations. We estimated the 75%-correct SF threshold, SF cutoff point (i.e., chance-level), and slope of the psychometric function for each location. We found higher SF estimates (i.e., better acuity) for the horizontal than the vertical meridian and for the lower than the upper vertical meridian. These asymmetries were most pronounced at the cardinal meridians and decreased gradually as the angular distance from the vertical meridian increased. This gradual change in acuity with polar angle reflected a shift of the psychometric function without changes in slope. The same pattern was found under binocular and monocular viewing conditions. These findings advance our understanding of visual processing around the visual field and help constrain models of visual perception.

## Introduction

**Figure 1.**
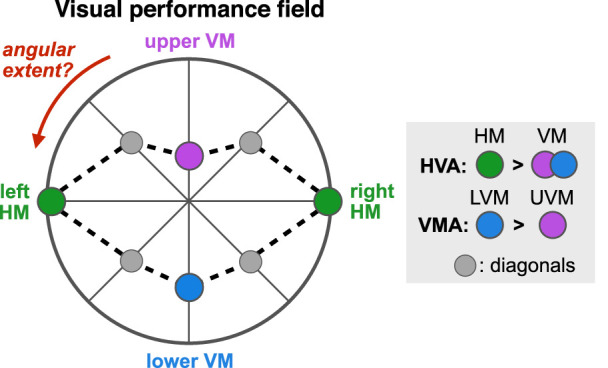
Graphic illustration of a canonical visual performance field (based on data from Carrasco et al., 2001). Each dot represents performance as a function of polar angle at a fixed eccentricity. The center of the polar plot corresponds to chance level, with highest performance typically observed along the horizontal meridian (HM; green), without differences between left and right hemifields. The horizontal-vertical anisotropy (HVA) depicts better performance in many tasks along the HM than the vertical meridian (VM). Moreover, performance is better at the lower VM (LVM; blue) than at the upper VM (UVM; purple), which is referred to as the vertical meridian asymmetry (VMA). Performance along the intercardinal (± 45°) meridians (gray) is usually similar, raising questions about the degree of visual performance fields as a function of polar angle.

Visual perception is not uniform across the visual field. Visual performance not only decreases as eccentricity increases (Cannon, 1985; Carrasco, Evert, Chang, & Katz, 1995; Rijsdijk, Kroon, & van der Wildt, 1980; Thibos, Cheney, & Walsh, 1987), but also varies across isoeccentric locations as a function of polar angle, a pattern referred to as *visual performance fields* (Altpeter, Mackeben, & Trauzettel-Klosinski, 2000; Cameron, Tai, & Carrasco, 2002; Carrasco, Talgar, & Cameron, 2001; Mackeben, 1999). Specifically, visual performance is better along the horizontal meridian (HM) than the vertical meridian (VM)—the *horizontal-vertical* *anisotropy* (HVA)—and better along the lower than the upper VM—the *vertical-meridian* *asymmetry* (VMA). Figure 1 illustrates the classic pattern of visual performance as a function of polar angle found in previous studies. Each dot represents performance at an isoeccentric location, with better performance indicated by points farther away from the center of the polar plot. The term *performance fields* was first introduced to describe performance asymmetries around the visual field (Altpeter et al., 2000; Mackeben, 1999), which were interpreted as differences in attentional performance. However, studies in which spatial attention has been manipulated, rather than inferred, have shown that attention modulates performance similarly across isoeccentric locations, without affecting the shape of performance fields (e.g., Carrasco et al., 2001, 2002; Purokayastha, Roberts, & Carrasco, 2020; Roberts, Ashinoff, Castellanos, & Carrasco, 2018; Roberts, Cymerman, Smith, Kiorpes, & Carrasco, 2016).

Performance fields are ubiquitous in visual perception, having been observed in numerous tasks, including those mediated by contrast sensitivity (e.g., Abrams, Nizam, & Carrasco, 2012; Baldwin, Meese, & Baker, 2012; Cameron et al., 2002; Carrasco et al., 2001; Corbett & Carrasco, 2011; Fuller, Rodriguez, & Carrasco, 2008; Himmelberg, Winawer, & Carrasco, 2020; Pointer & Hess, 1989; Rosen, Lundstrom, Venkataraman, Winter, & Unsbo, 2014; Rovamo & Virsu, 1979), spatial resolution (Altpeter et al., 2000; Carrasco et al., 2002; De Lestrange-Anginieur & Kee, 2020; Greenwood, Szinte, Sayim, & Cavanagh, 2017; Montaser-Kouhsari & Carrasco, 2009; Nazir, 1992; Talgar & Carrasco, 2002), color hue (Levine & McAnany, 2005), motion (Fuller & Carrasco, 2009; Lakha & Humphreys, 2005; Levine & McAnany, 2005), spatial crowding (Greenwood, Szinte, Sayim, & Cavanagh, 2017; Petrov & Meleshkevich, 2011; Wallis & Bex, 2012), saccadic precision and spatial localization (Greenwood et al., 2017), saccade latency (Petrova & Wentura, 2012; Greene, Brown, & Dauphin 2014; Greenwood et al., 2017), peak saccade velocity (Greenwood et al., 2017), and speed of information accrual (Carrasco, Giordano, & McElree, 2004). Performance asymmetries are retinotopic, shifting in line with the retinal location of the stimulus rather than its location in space (Corbett & Carrasco, 2011), and pervasive, emerging regardless of stimulus orientation or display luminance (Carrasco et al., 2001). Furthermore, performance fields become more pronounced as eccentricity (Baldwin et al., 2012; Carrasco et al., 2001; Himmelberg et al., 2020; Rijsdijk et al., 1980), spatial frequency (Cameron et al., 2002; Carrasco et al., 2001; Himmelberg et al., 2020; Liu, Heeger, & Carrasco, 2006; Rijsdijk et al., 1980), and set size (Carrasco et al., 2001; Lakha & Humphreys, 2005; von Grunau & Dube, 1994) increase. Note that although performance fields usually become more pronounced as set size increases, visual asymmetries are present when target stimuli are presented alone (e.g., Baldwin et al., 2012; Cameron et al., 2002; Carrasco et al., 2001; Carrasco, Williams, & Yeshurun, 2002; Fuller & Carrasco, 2009; Rijsdijk et al., 1980). Performance asymmetries in perceived spatial frequency (SF) are also maintained in visual working memory (Montaser-Kouhsari & Carrasco, 2009).

Given the well-established asymmetries at the four cardinal locations, it is important to characterize whether and how the HVA and VMA extend away from the cardinal locations by measuring visual processing as a function of polar angle. The question is whether visual asymmetries are restricted to the cardinal meridians or whether (and how far) they extend into the upper and lower hemifields. In this context, the *HVA* corresponds to the HM-VM asymmetry observed at 0° angular distance from the VM. The *angular extent of the HVA* corresponds to the difference in performance between the HM and isoeccentric stimuli placed away from the VM, as a function of the angular distance from the VM (from 0° to 90° polar angle). Similarly, the *VMA* corresponds to the upper VM (UVM)–lower VM (LVM) asymmetry observed at 0° angular distance from the VM, and the *angular extent of the VMA* refers to the asymmetry between upper and lower isoeccentric locations measured as a function of the angular distance from the VM.

A previous study from our lab (Abrams et al., 2012) characterized the angular extent of these visual asymmetries for contrast sensitivity—a fundamental visual dimension—and showed that they are most pronounced at the VM and decrease gradually as the angular distance from the VM increases. This finding is consistent with previous findings showing similar performance at intercardinal (± 45° polar angle) locations (e.g., Altpeter et al., 2000; Baldwin et al., 2012; Cameron et al., 2002; Carrasco et al., 2001; Corbett & Carrasco, 2011; Fuller et al., 2008; Liu et al., 2006; Mackeben, 1999; Nazir, 1992; Talgar & Carrasco, 2002). The gray points representing equal performance at 45° polar angle in Figure 1 illustrate this finding**.** Given that the magnitude of performance fields becomes more pronounced for higher SFs and for further eccentricities (e.g., Baldwin et al., 2012; Cameron et al., 2002; Carrasco et al., 2001; Himmelberg et al., 2020; Liu et al., 2006), our primary goal here was to investigate whether and how far these visual asymmetries change away from the VM in terms of visual acuity.

**Figure 2.**
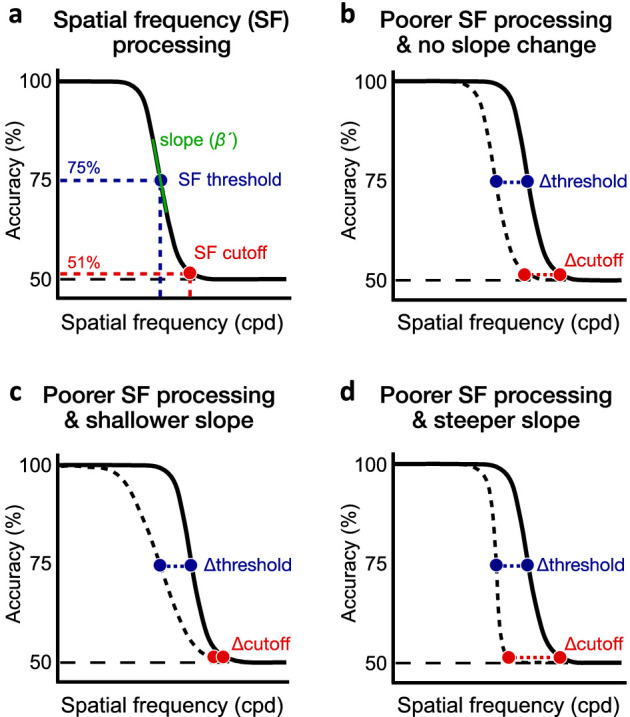
Spatial frequency (SF) processing. (a) Performance in SF discrimination decreases as stimulus SF increases. For each polar angle location, we estimated the 75%-correct SF threshold (blue dot) and SF cutoff (red dot) corresponding to the SF at which participants were near chance level (i.e., 51% correct). We also estimated the slope (β) of the psychometric function, which was converted into the maximum slope estimate (β´) (see Methods). (b) Differences in SF processing between two locations (e.g., UVM and LVM) could reflect a shift of the psychometric function without a change in slope. Such change would result in a similar difference in SF threshold and SF cutoff. (c, d) Asymmetries in SF processing could also be characterized by a change in the slope of the psychometric function. Relative to a similar change in SF threshold in both panels, a (c) shallower or (d) steeper slope would result in the change in SF cutoff to be less or more pronounced, respectively.

Spatial resolution—our ability to discriminate fine patterns—is a fundamental dimension of visual perception. In the present study, we assessed performance fields in spatial resolution by measuring visual acuity as a function of polar angle. We hypothesized that the angular extent of asymmetries in visual acuity will be similar to that of contrast sensitivity—that is, most pronounced at the VM and decaying gradually as the angular distance from the VM toward the HM increases. Participants were asked to discriminate the orientation of high-contrast grating stimuli varying in SF, presented at 1 of 24 isoeccentric locations (at steps of 15° polar angle) at 10 deg eccentricity. We characterized performance fields in grating acuity in terms of 75%-correct SF thresholds (i.e., the SF at which observers can reliably discriminate stimulus orientation). In addition, we estimated SF cutoffs (i.e., the SF at which observers’ performance drops to chance level) as a secondary measure (Figure 2a). Finally, by estimating the full SF psychometric function, we were also able to assess whether performance fields are characterized by a shift of the psychometric function without a change in its slope (Figure 2b) or whether performance fields also reflect changes in the slope of the psychometric function (Figures 2c,d). Differences in slope would differently affect SF threshold and SF cutoff estimates and indicate differences in the reliability of sensory SF estimates across isoeccentric locations.

Another goal of the present study was to assess whether variations in *horizontal disparity*—the difference in the azimuth between the images formed by the two eyes—across the visual field could be a potential source of visual performance fields. This hypothesis is based on two main findings: (1) Horizontal disparity is absent from the HM but varies along the VM: Stimuli presented above the fixation point have *uncrossed disparity* and are perceived as further away from fixation, whereas stimuli below fixation have *crossed disparity* and appear to be closer to fixation. This pattern has been shown both behaviorally in humans (Helmholtz, 1925; Hibbard & Bouzit, 2005; Sprague, Cooper, Reissier, Yellapragada, & Banks, 2016; Sprague, Cooper, Tosic, & Banks, 2015) and neurophysiologically in monkeys (Sprague et al., 2015). (2) Blur caused by disparity covers more area as stimulus eccentricity increases (Sprague et al., 2016). Given that these two factors increase with eccentricity, horizontal disparity could be a contributing source to the HVA and VMA and affect their magnitude. To investigate this possibility, we tested whether the HVA and VMA differ between binocular and monocular viewing conditions, as disparity is nonexistent for the latter.

## Methods

### Observers

Fourteen observers (10 females; age: 25.5 ± 5.5 years, age range: 23–35 years) with normal or corrected-to-normal vision participated in the binocular condition. All but one (author AB) were naive with respect to the purpose of this study. Eight were experienced psychophysical observers and the other six were not. Seven of them (6 females; age: 25.1 ± 4.1 years, age range: 23–35 years) also participated in the monocular experiment, in which only their dominant eye was tested (5/7 observers were right-eye dominant). Observers were paid $10/hour. The Institutional Review Board at New York University approved the experimental procedures, and all observers gave informed consent.

### Apparatus

All stimuli were generated and presented using MATLAB (MathWorks, Natick, MA, USA) and the Psychophysics Toolbox (Kleiner, Brainard, & Pelli, 2007) on a CRT monitor (1,600 × 1,200 screen resolution; 60 Hz; 53 cd/m^2^ background luminance). Observers viewed the display at a distance of 57 cm with their head stabilized by a chinrest. An eye-tracker system (EyeLink 1000, SR Research, Ottawa, ON, Canada) was located in front of the observer to track eye position.

### Stimuli

**Figure 3.**
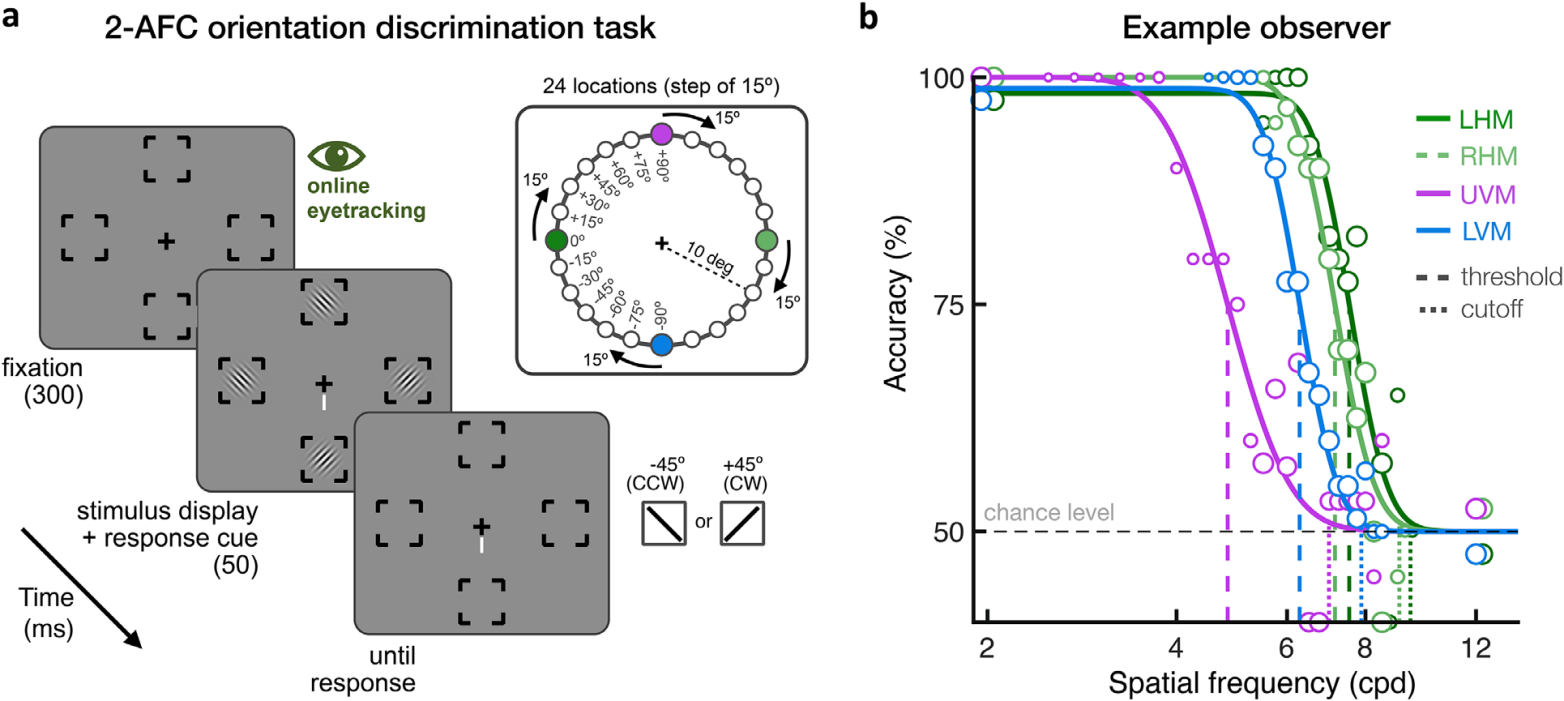
(a) Trial sequence. Observers were asked to maintain fixation at the center of the screen, which was ensured using online eye-tracking. In a given session, grating stimuli were presented at four isoeccentric (10 deg eccentricity) locations. Observers were asked to report the orientation of the target stimulus at the location indicated by the response cue. Spatial frequency (SF) varied across trials. A total of 24 isoeccentric locations were tested across separate blocks by rotating the angular position of the 4 stimulus locations by 15°. The size of the placeholders, fixation point, and response cue have been enlarged for illustration purposes. (b) Example observer. Psychometric functions for one observer at the four cardinal locations (LHM/RHM = left/right horizontal meridian; UVM/LVM = upper/lower vertical meridian). Vertical dashed lines indicate the 75%-correct SF thresholds, and the dotted lines indicate SF cutoff estimates. The SF range used for each observer and location was adjusted between sessions to capture the dynamic range of the psychometric function. The size of each data point varies with the number of trials collected at each SF level.

As illustrated in Figure 3a, the visual display consisted of four components: a black fixation cross (0.2 × 0.2 deg) presented at the center of the screen, four placeholders, four stimuli, and a response cue. The fixation cross and the four placeholders were always present across all frames of each trial to eliminate spatial uncertainty. The placeholders were centered at the four isoeccentric, equidistant stimulus locations (10 deg eccentricity), each separated by 90° polar angle. Each placeholder was composed of four corners (0.25 deg line length) delimiting a virtual square (3.5 × 3.5 deg). In a given block, the axes of the four placeholders were rotated clockwise from the vertical meridian by 0°, 15°, 30°, 45°, 60°, 75°, or 90° polar angle. This design enabled measuring orientation discrimination performance as a function of SF at 24 evenly spaced isoeccentric locations in the periphery, with only four locations tested simultaneously in a given block. Stimuli were suprathreshold (100% contrast) grating patches delimited by a raised-cosine envelope (2.5 deg diameter) and oriented ± 45° from vertical. Note that stimuli presented at adjacent isoeccentric locations, which were tested on different blocks, would have not overlapped with each other as the distance between the centers of two adjacent locations was 2.6 deg and the radius of each stimulus was 1.25 deg. In a given trial, the four stimuli had the same SF, which varied from trial to trial from 3 to 12 cpd, in 0.25-cpd steps. The response cue consisted of a white line presented next to one arm of the fixation cross to indicate which one of the four possible stimulus locations was the target location. The stimuli and the response cue were presented simultaneously to eliminate spatial uncertainty about the target location (e.g., Ling & Carrasco, 2006; Ling, Liu, & Carrasco, 2009; Lu & Dosher, 2000).

### Procedure

Figure 3a depicts the trial sequence. Each trial started with a 300-ms fixation period followed by the 50-ms presentation of four grating stimuli. Along with stimulus presentation, a response cue pointing to the target location was presented at fixation until a response was made. Observers were asked to report the orientation (± 45° counterclockwise or clockwise off vertical) of the stimulus presented at the target location by pressing either the left (counterclockwise) or down (clockwise) arrow keys. Auditory feedback was provided after each response, with either a high-pitched or low-pitched beep denoting a correct or incorrect response, respectively. The start of each trial was contingent on stable fixation (1.5 deg radius around the central fixation), which was ensured during the full trial sequence using online eye-tracking. Each block consisted of 300 trials, corresponding to 5 trials for each of 15 different SF values for each of the four tested locations within a block. The range of SF values always contained 2 and 12 cpd, to ensure a good estimation of the lower and upper asymptotes, in addition to 13 SF values centered on a given SF, with equal steps of 0.25 cpd. Based on pilot data, all observers were initially tested using a range of SF values centered at 7 cpd. The central SF value for each location was then adjusted in subsequent blocks, if needed, to ensure that the SF range was centered on the dynamic range for each observer and for each location.

Each observer completed five or six 1-hr sessions for a total of 9,107 ± 1,174 trials on average, consisting of 621 ± 85 trials on average at each of the four cardinal locations (two VM locations and two HM locations), and of 331 ± 48 trials on average at each of the 20 noncardinal locations. The four cardinal locations were tested twice more to increase power at these critical locations and to equate the number with the other locations, once we combined data at mirror locations on the left and right hemifields. Indeed, consistent with other studies (e.g., Abrams et al., 2012; Baldwin et al., 2012; Carrasco et al., 2001; Greene, Brown, & Dauphin, 2014; Petrov & Meleshkevich, 2011; Purokayastha et al., 2020), we did not find differences between the left and right hemifields. The same procedure was used in the monocular viewing condition (7,500 ± 917 trials collected, on average), except that the observer's nondominant eye was covered.

### Analysis

Psychometric functions were fit to the data using the Palamedes Toolbox (Prins & Kingdom, 2018). For each location, a cumulative normal distribution function was fit to the data using maximum likelihood estimation, with the function given as

(1) $$\begin{array}{c} \begin{array}{c} \;\;\;\;\;\;\;\;\;\;\;\;\;f\left(SF\right)=\gamma +\left(1-\gamma -\lambda \right)*\;\frac{\beta }{\sqrt{2\pi }}*\int_{-\infty }^{SF}exp\left(-\frac{\beta^{2}{\left(SF-\alpha \right)}^{2}}{2}\right),\; \end{array} \end{array}$$

in which *f*(*SF*) is the performance as a function of stimulus SF (in log cpd), α is the location parameter, β is the slope, and γ and λ are the lower and upper asymptotes, respectively. The lower asymptote γ was fixed to chance level (50% correct). The SF range was flipped in log-space when fitting the data to reflect the increasing psychometric function. For each location, we estimated the *SF threshold* (i.e., 75% correct), as well as the *SF cutoff* (i.e., 51% accuracy), and the *slope* of the psychometric function. As the slope value (β) depends on the psychometric function used to fit the data, the slope estimate (β) was converted into the maximum slope (β′) using the following equation (i.e., Equation 18 from Strasburger, 2001a):

(2) $$\beta^{'}=\left(\frac{1-\gamma }{\sqrt{2\pi }}\right)*\;\beta$$

Figure 3b shows the psychometric functions at the cardinal locations for an example observer. After collecting data at 24 locations for all observers, we tested the left-right difference in performance and found it to be nonsignificant. Thus, we also collapsed the data in the right hemifield to the horizontally corresponding position in the left hemifield to increase the number of trials at each location and refit the new data using the method described above. Log-value estimates were used for statistical analysis. To assess the HVA and VMA, repeated-measures analyses of variance (ANOVAs) were used to assess differences in SF estimates at the four cardinal locations (left HM, right HM, upper VM, and lower VM), as well as differences between viewing conditions (monocular vs. binocular). In all cases in which Mauchly's test of sphericity indicated a violation of the sphericity assumption, Greenhouse-Geisser–corrected values were used. Partial eta-square (η^2^_p_) and Cohen's *d* are reported as an estimate of effect size for the ANOVAs and paired *t*-tests, respectively. To characterize asymmetries in acuity as a function of polar angle, we used linear mixed-effects models to predict SF estimates based on the angular distance from the VM (0°, 15°, 30°, 45°, 60°, 75°, and 90°), visual field (upper vs. lower), and viewing condition (monocular vs. binocular), whereas differences between participants were considered a random effect. Scatterplots of individual estimates, along with multiple linear regression equations and adjusted *R*^2^, are reported for the linear mixed-effects models. SF estimates are also reported in physical units (e.g., SF threshold in cpd). Note that there was no effect of participant's biological sex, with neither significant main effects nor interactions (all *p* values > 0.1) for any of the analyses reported below.

## Results

### Visual asymmetries across cardinal locations: HVA and VMA

**Figure 4.**
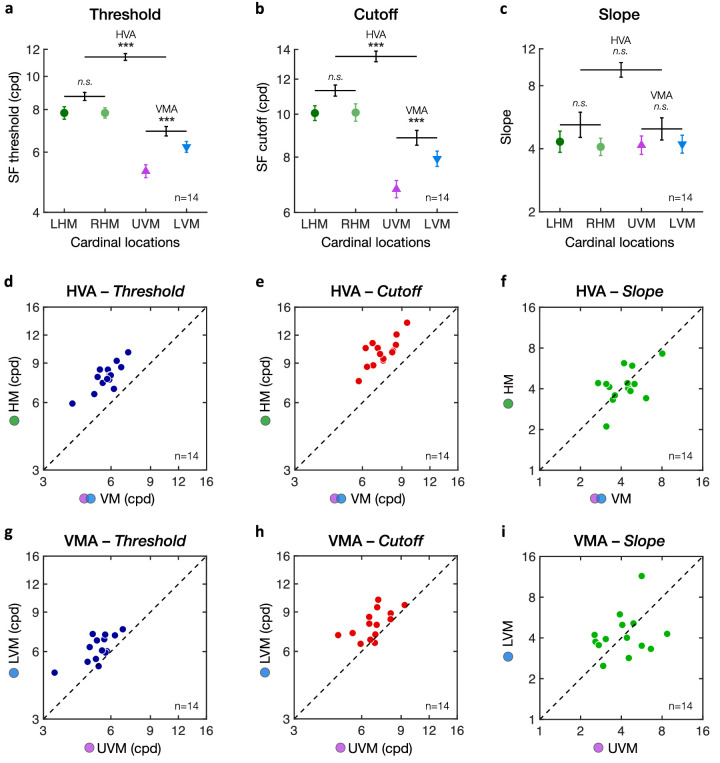
Horizontal vertical anisotropy (HVA) and vertical meridian asymmetry (VMA). Averaged binocular (a) SF threshold, (b) SF cutoff, and (c) slope estimates at each of the four cardinal locations (LHM = left horizontal meridian; RHM = right horizontal meridian; UVM = upper vertical meridian; LVM = lower vertical meridian). There was no difference between the LHM and RHM. The HVA corresponds to the difference between the HM (LHM and RHM combined) and VM (LVM and UVM combined). The VMA corresponds to the difference between the LVM and UVM. Error bars in panels a to c correspond to ± 1 *SEM* for each of the cardinal data points. Horizontal lines reflect comparisons between the LHM and RHM, between the HM and VM (i.e., HVA), and between the UVM and LVM (i.e., VMA), with error bars representing ± 1 *SE* of the mean difference. *p < 0.05, **p < 0.01, ***p < 0.001. (d–f) Scatterplots of individual participants' HVA for (d) threshold, (e) cutoff, and (f) slope estimates. (g–i) Scatterplots of individual participants' VMA for (g) threshold, (h) cutoff, and (i) slope estimates. Dots above the diagonal line indicate participants showing typical HVA and VMA patterns, which are observed for SF threshold and SF cutoff estimates but not for slope.

Figure 4 shows the performance at the four cardinal locations (left and right HM, upper and lower VM) averaged across observers, demonstrating a clear HVA and VMA in both SF threshold (Figure 4a) and SF cutoff (Figure 4b). One-way repeated-measures ANOVAs revealed significant differences between cardinal locations for both SF thresholds (*F*(3, 39) = 79.25, *p* < 0.001, η^2^_p_ = .86) and SF cutoffs (*F*(3, 39) = 59.35, *p* < 0.001, η^2^_p_ = .82). No difference in the slope estimates of the psychometric functions was observed (Figure 4c; *F*(3, 39) < 1). These results indicate that both the HVA and VMA reflect shifts of the psychometric functions without change in shape (Figure 2b). As expected, there was no difference between the left and right HM for both SF thresholds (LHM: 7.82 ± 1.23 cpd; RHM: 7.82 ± .99 cpd) and SF cutoffs (LHM: 10.06 ± 1.46 cpd; RHM: 10.09 ± 1.77 cpd). The HVA reflected higher acuity along the horizontal meridian (LHM and RHM) than along the vertical meridian (UVM and LVM) for both SF thresholds (HM: 7.82 ± 1.06 cpd; VM: 5.73 ± 0.75 cpd; *t*(13) = 13.73, *p* < 0.001, Cohen's *d* = 3.67) and SF cutoffs (HM: 10.07 ± 1.52 cpd; VM: 7.33 ± 1.06 cpd; *t*(13) = 11.53, *p* < 0.001, Cohen's *d* = 3.08). Moreover, characteristic of the VMA, performance at the LVM was significantly better than at the UVM, for both SF threshold (LVM: 6.22 ± 0.84 cpd; UVM: 5.28 ± 0.79 cpd; *t*(13) = 5.14, *p* < 0.001, Cohen's *d* = 1.37) and SF cutoffs (LVM: 7.93 ± 1.21 cpd; UVM: 6.77 ± 1.14 cpd; *t*(13) = 4.11, *p* < 0.001, Cohen's *d* = 1.10).

**Figure 5.**
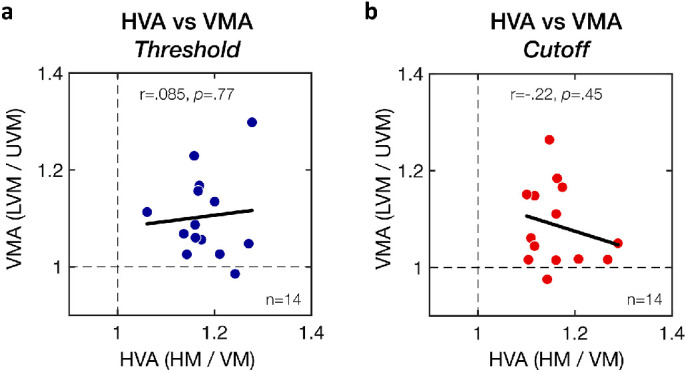
Lack of correlation between the HVA and VMA. HVA and VMA ratios estimated from (a) SF threshold estimates or (b) SF cutoff estimates. Each data point corresponds to an individual participant's HVA and VMA ratios, with the solid black lines corresponding to Pearson correlations.

To examine group variability, we plotted individual estimates of the HVA and VMA for SF threshold (Figures 4d,g), SF cutoff (Figures 4e,h), and slope (Figures 4f,i) estimates. Each dot represents an individual estimate, with the dashed diagonal line indicating equal performance. All observers showed a clear HVA (Figures 4d–f) and VMA (Figures 4g–i) for SF threshold and SF cutoff estimates, with all individual estimates (except one for the VMA) being above the diagonal line. Slope estimates were distributed around the diagonal line, indicating no consistent changes in slope for either the HVA or VMA. Moreover, whereas all participants showed both HVA and VMA, there was no significant correlation between these two types of visual asymmetries (Figure 5; SF threshold: *r* = .085, *p* = 0.77; SF cutoff: *r* = –.22, *p* = 0.45).

### No difference between left and right hemifields

**Figure 6.**
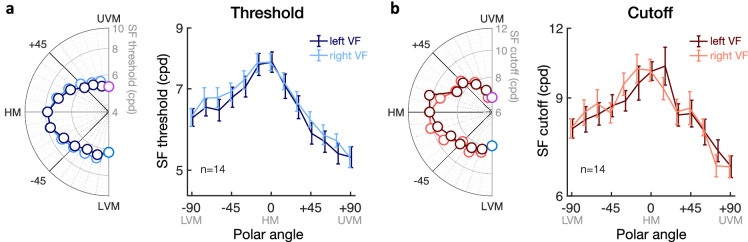
No left-right hemifield difference. Changes in (a) SF threshold and (b) SF cutoff as a function of polar angle for the left and right hemifields. Polar plots of hemifields (left panels) show group-averaged SF estimates as a function of polar angle for the left and right hemifield locations separately (the data points corresponding to the UVM and LVM are color-coded as in Figure 1). Right panels show the same data with error bars corresponding to ± 1 *SEM*. No difference was observed between the left and right visual field (VF) locations. The asymmetry with polar angle between lower (−90° to 0°) and upper (0° to +90°) VF locations is characteristic of the VMA (HM = horizontal meridian; UVM/LVM = upper and lower vertical meridians).

There was no significant left-right difference at the HM. We assessed whether this was also the case when comparing all tested locations. A two-factor repeated-measures ANOVA (2 hemifields × 11 non-VM locations) showed no significant difference between the left and right hemifields for either SF threshold (Figure 6a; *F*(1, 13) = 2.55, *p* = 0.134, η^2^_p_ = .16), SF cutoff (Figure 6b; *F*(1, 13) < 1), or slope (*F*(1, 13) < 1). We found a substantial effect of polar angle on SF threshold (*F*(3.8, 49.2) = 53.72, *p* < 0.001, η^2^_p_ = .81) and SF cutoff (*F*(10, 130) = 15.70, *p* < 0.001, η^2^_p_ = .55) estimates but only a marginal effect on slope (*F*(3.7, 48.1) = 2.39, *p* = 0.068, η^2^_p_ = .16). Importantly, there was no interaction between polar angle location and left-right hemifields (all *p* values > 0.1). Given the absence of left-right hemifield difference as a function of polar angle, we reanalyzed each polar angle location after collapsing the data across hemifields for simplicity of analysis and clarity of illustration.

### Gradual decrease in visual asymmetries with increasing angular distance from the vertical meridian

**Figure 7.**
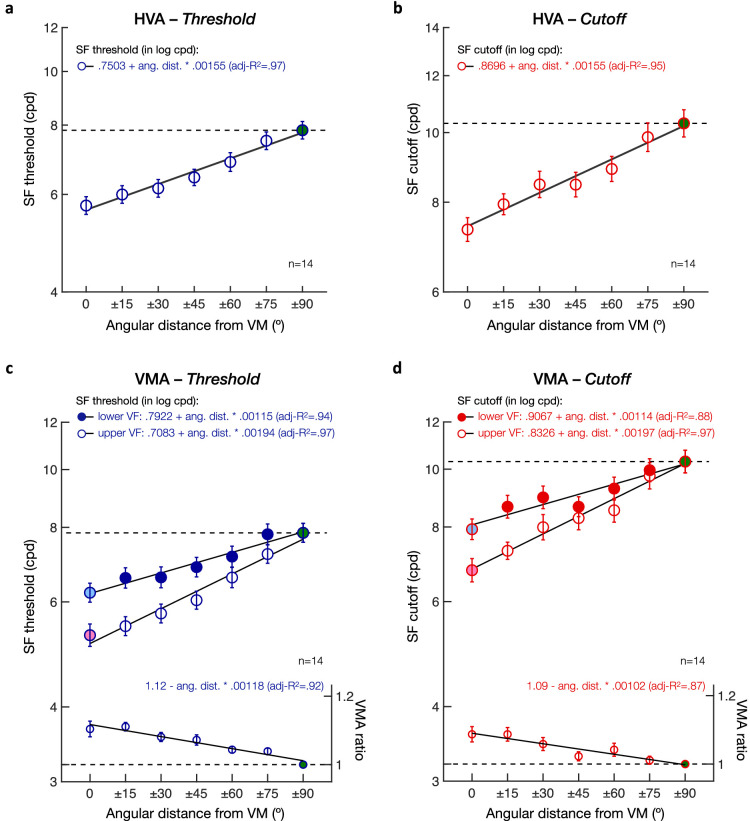
Angular extent of asymmetries in visual acuity. Group-averaged (a, c) SF threshold and (b, d) SF cutoff estimates plotted as a function of the angular distance from the vertical meridian (VM). Dashed line represents the value at the horizontal meridian (HM; green filled dot). (a, b) Horizontal vertical anisotropy (HVA). SF estimates were averaged across upper and lower hemifields, with the difference from the HM at 0° angular distance from the VM corresponding to the HVA. (c, d) Vertical meridian asymmetry (VMA). SF estimates plotted separately for the upper VF (open circles) and lower VF (filled circles), with the upper-lower difference at 0° angular distance from the VM corresponding to the VMA. VMA ratios plotted at the bottom of panels c and d were computed by dividing the lower by the upper visual field estimates. Adjusted *R*^2^ values indicate the goodness of fit of linear regression equations. Error bars correspond to ± 1 *SEM*.

First, we assessed whether and how the HVA extends from the VM. Figure 7 shows SF threshold (Figure 7a) and SF cutoff (Figure 7b) estimates, averaged over upper and lower hemifields, plotted as a function of the angular distance from the VM (from 0° to ± 90°, in 15° steps). We used linear mixed-effects models to predict SF estimates based on the angular distance from the VM, with participants as a random effect. We found that both SF threshold and SF cutoff estimates increased as the angular distance from the VM increased. Participants’ SF threshold (in log cpd) was equal to .7503 + .00155 * angular distance (intercept: *t*(96) = 48.53, *p* < 0.001, CI [.7196, .7809]; angular distance: *t*(96) = 13.09, *p* < 0.001, CI [.0013, .0018]). Similarly, participants’ SF cutoff (in log cpd) was equal to .8696 + .00155 * angular distance (intercept: *t*(96) = 60.32, *p* < 0.001, CI [.8410, .8983]; angular distance: *t*(96) = 13.02, *p* < 0.001, CI [.0013, .0018]). No difference in the slope of the psychometric functions was observed as a function of angular distance (intercept: *t*(96) = 26.53, *p* < 0.001, CI [.5352, .6218]; angular distance: *t*(96) = .39, *p* = 0.700, CI [–.0007, .0011]). In other words, SF estimates at isoeccentric locations were predicted to linearly increase from the VM to the HM, from 5.73 to 7.82 cpd for SF threshold and from 7.33 to 10.07 cpd for SF cutoff. The similar slope of the linear regression equations for SF threshold and SF cutoff estimates is consistent with the lack of change in the psychometric function slope with polar angle. In sum, the asymmetry observed between the HM and VM (i.e., HVA) is not restricted to the VM but rather reflects a linear change in visual acuity between the HM and VM, without differences in the slope of the psychometric function.

**Figure 8.**
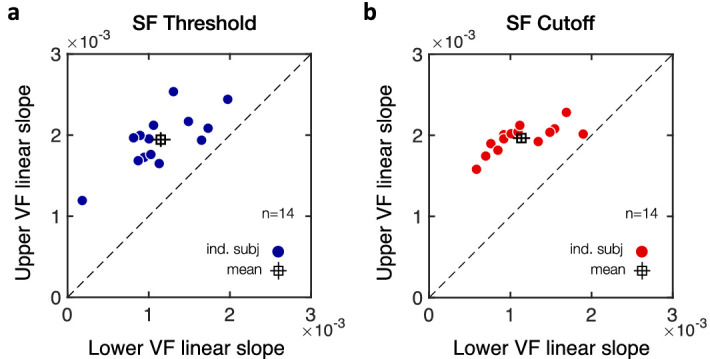
Individual linear slope estimates from the linear mixed-effects models. Scatterplots of individual linear slope estimates with angular distance from the VM show a steeper linear slope in the upper than lower visual field (VF) in all participants (*n* = 14), for both (a) SF threshold and (b) SF cutoff estimates. Filled circles correspond to individual participants and the open square symbol to the mean ± 1 *SEM*.

Next, we assessed whether and how the VMA extends from the VM for both SF threshold (Figure 7c) and SF cutoff (Figure 7d) estimates. Specifically, we measured how the gradual change in SF estimates with angular distance from the VM differed between lower and upper visual field locations. We used linear mixed-effects models to predict both SF threshold and SF cutoff estimates, including the angular distance from the VM and visual field (i.e., upper vs. lower VF) as predictors and participants as a random effect. As expected, SF threshold estimates linearly increased with the angular distance from the VM (intercept: *t*(192) = 48.53, *p* < 0.001, CI [.7198, .7808]; angular distance: *t*(192) = 13.09, *p* < 0.001, CI [.0013, .0018]). Consistent with the presence of a VMA, SF threshold estimates were higher in the lower VF than in the upper VF at the VM (visual field: *t*(192) = 7.95, *p* < 0.001, CI [.0315, .0523]), but increased at a faster rate in the upper VF than in the lower VF with increasing angular distance from the VM (angular distance * visual field interaction: *t*(192) = 5.87, *p* < 0.001, CI [.0003, .0005]). Participants’ SF threshold (in log cpd) was equal to .7083 + .00194 * angular distance in the upper VF and to .7922 + .00115 * angular distance in the lower VF. A similar pattern was observed for SF cutoff estimates, with participants’ SF cutoff (in log cpd) being equal to .8326 + .00197 * angular distance in the upper VF and to .9067 + .00114 * angular distance in the lower VF (intercept: *t*(192) = 60.13, *p* < 0.001, CI [.8411, .8982]; angular distance: *t*(192) = 12.76, *p* < 0.001, CI [.0013, .0018]; visual field: *t*(192) = 5.61, *p* < 0.001, CI [.0240, .0501]; angular distance * visual field interaction: *t*(192) = 4.05, *p* < 0.001, CI [.0002, .0006]). Neither angular distance nor visual field had an effect on the psychometric slope estimates (*p* > 0.1 and CI includes 0 for angular distance, visual field, and the interaction). These results indicate that SF estimates linearly increase between the VM and HM but do so at a faster rate in the upper VF, resulting in a gradual decrease in the upper-lower asymmetry. This pattern was observed in all participants, with higher individual linear slope estimates as a function of the angular distance from the VM in the upper VF than in the lower VF for both SF threshold (Figure 8a) and SF cutoff (Figure 8b) estimates.

### Performance fields become more pronounced with increasing stimulus SF

**Figure 9.**
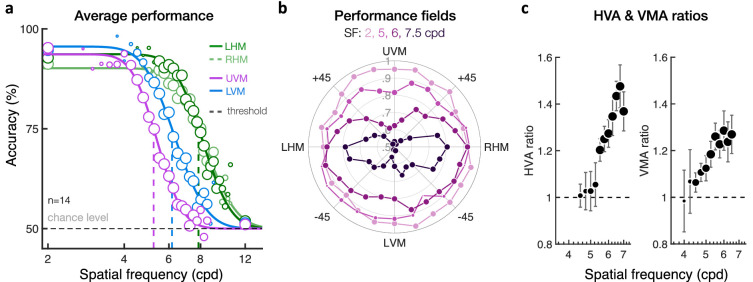
Impact of stimulus SF on performance fields. (a) Group-averaged orientation discrimination performance plotted as a function of the stimulus SF at the four cardinal locations (LHM/RHM = left/right horizontal meridian; UVM/LVM = upper/lower vertical meridian). Performance decreases similarly with increasing SF at the LHM and RHM locations, resulting in similar psychometric functions along the HM. Relative to the HM, performance at the VM is worse (i.e., HVA). Moreover, performance at the UVM location is poorer than at the LVM location (i.e., VMA). These asymmetries in SF processing reflected shifts of the psychometric functions without change in slope. Marker size indicates the number of participants averaged for each data point, which was restricted to a minimum of 4 out of the 14 participants. (b) Polar plot showing group-averaged performance as a function of the stimulus polar angle and SF, with the center of the polar plot corresponding to chance level (50% accuracy). Asymmetries at isoeccentric locations become more pronounced as stimulus SF increases. (c) Both the HVA and VMA performance ratios increase with stimulus SF. Each data point is the average performance ratio (± 1 *SEM*) computed at different stimulus SF within the dynamic range of the psychometric functions. Marker size indicates the number of participants averaged for each data point (varying from 4 to 14 participants).

Our results provide additional evidence of how performance fields vary with stimulus SF. Both the HVA and VMA can be described by a shift of the psychometric function without change in slope (Figure 9a). We found that performance fields become more pronounced as stimulus SF increases (Figure 9b). At low SFs, performance is high and varies slightly with polar angle. As stimulus SF increases, performance decreases but does so faster for stimuli presented closer to the VM than near the HM, resulting in the HVA. Performance also decreases faster for stimuli presented at the lower VM than at the upper VM, resulting in the VMA. We estimated HVA and VMA accuracy ratios as a function of stimulus SF (Figure 9c). The SF range used was chosen to match the dynamic range of the psychometric functions where performance ratios can be estimated (i.e., where performance is neither at ceiling nor at chance level and where enough participants were tested). Both the HVA and VMA ratios increased with stimulus SF (Figure 9c). This pattern was attenuated when the angular distance from the VM increased.

### Similar performance fields under monocular and binocular viewing conditions

**Figure 10.**
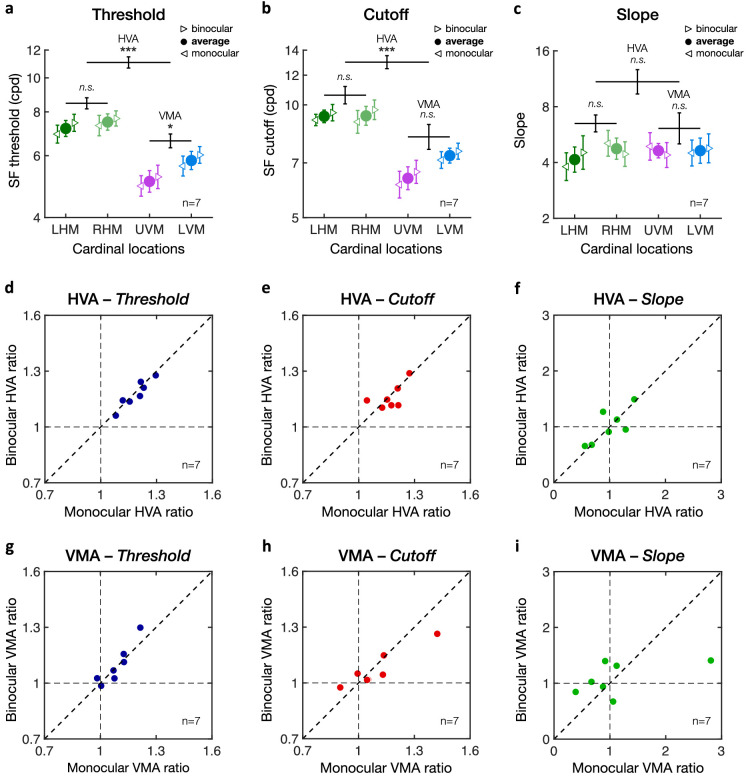
HVA and VMA under binocular and monocular viewing conditions. Averaged (a) SF threshold, (b) SF cutoff, and (c) slope estimates at each of the four cardinal locations (LHM/RHM = left/right horizontal meridian; UVM/LVM = upper/lower vertical meridian). Leftward and rightward triangles correspond to the monocular and binocular viewing condition, respectively. Filled circles correspond to average estimates across viewing conditions. Error bars correspond to ± 1 *SEM*. Horizontal lines reflect comparisons between the LHM and RHM, between the HM and VM (i.e., HVA), and between the UVM and LVM (i.e., VMA) for the combined binocular-monocular average data points, with error bars representing ± 1 *SE* of the mean difference. *p < 0.05, **p < 0.01, ***p < 0.001. (d–f) Scatterplots of individual participants’ HVA ratios (HM/VM) for (a) SF threshold, (b) SF cutoff, and (c) slope estimates. (g–i) Scatterplots of individual participants’ VMA ratios (LVM/UVM) for (g) SF threshold, (h) SF cutoff, and (i) slope estimates.

**Figure 11.**
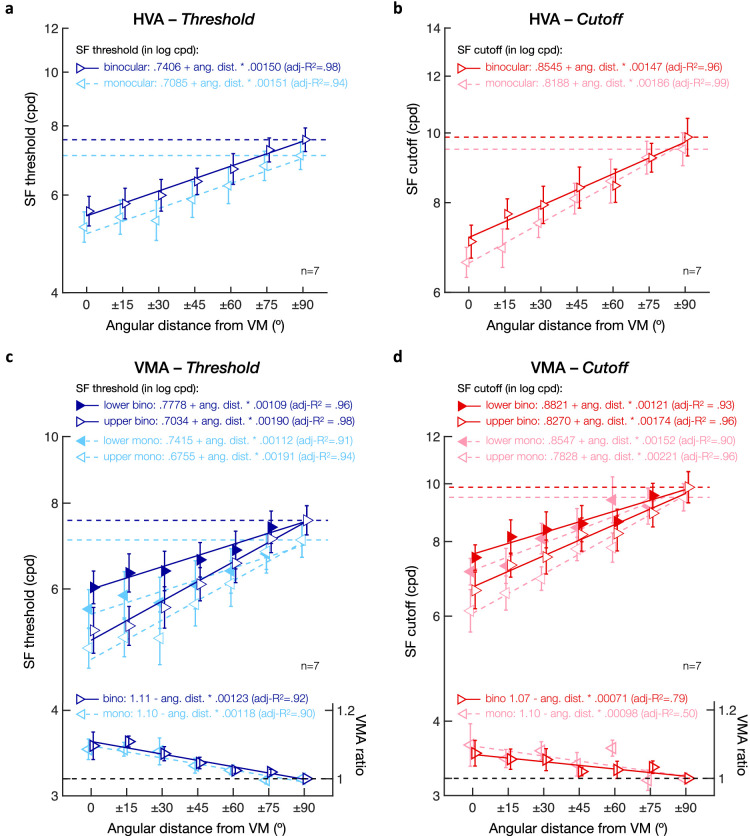
Angular extent of visual asymmetries under monocular and binocular viewing conditions. (a, b) SF threshold and (b, d) SF cutoff estimates plotted as a function of the angular distance from the vertical meridian (VM). (a, b) HVA extent. SF estimates were computed for monocular (leftward triangles; dashed lines) and binocular (rightward triangles; solid lines) viewing conditions by averaging values at upper and lower visual field locations. (c, d) VMA extent. SF estimates for monocular (leftward triangles; dashed lines) and binocular (rightward triangles; solid lines), plotted separately for upper (open triangles) and lower (filled triangles) visual field locations. VMA ratios at the bottom of panels c and d were computed by dividing the lower by the upper visual field estimates for monocular and binocular viewing conditions separately. Linear regression equations and adjusted *R*^2^ are provided for each linear fit. Error bars correspond to ± 1 *SEM*. Horizontal dashed lines represent values at the horizontal meridian.

**Figure 12.**
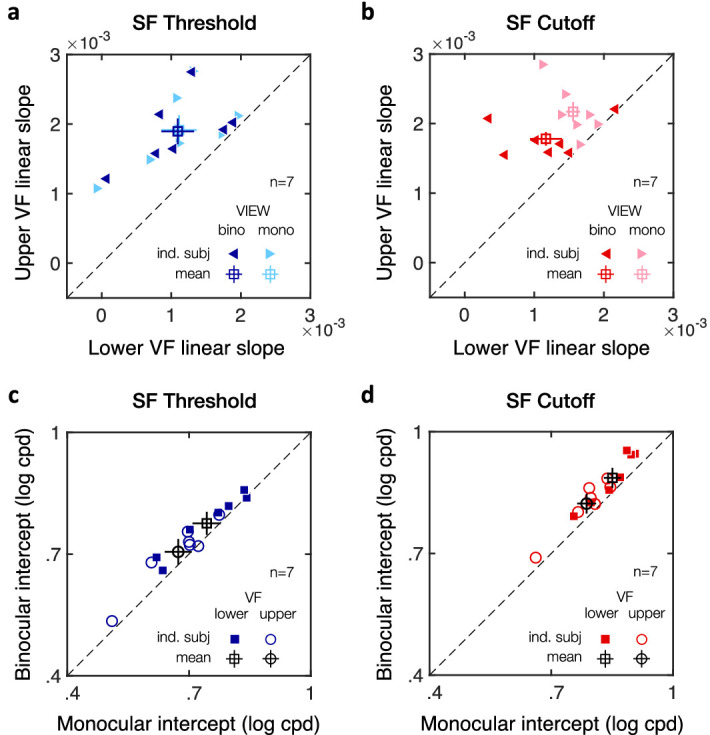
Individual estimates from the linear mixed-effects models. (a, b) Scatterplots of individual linear slope estimates with angular distance from the VM show steeper linear slope in the upper than lower visual field (VF), for both (a) SF threshold and (b) SF cutoff estimates. (c, d) Scatterplots of individual intercept estimates show higher SF intercepts under binocular than monocular viewing condition, for both (c) SF threshold and (d) SF cutoff estimates. Filled symbols correspond to individual participants (*n* = 7) and the open square symbols to the mean ± 1 *SEM*.

Figures 10 to 12 show monocular and binocular SF estimates for the seven observers tested under both monocular and binocular viewing conditions. Monocular performance was measured only for the observers’ dominant eyes (i.e., right eye for five observers and left eye for the other two). We did not observe differences in SF estimates between the nasal and temporal retinal hemifields, which usually emerge further in the periphery, beyond 10 deg eccentricity (Harvey & Pöppel, 1972; Pöppel & Harvey, 1973). Similar to the results described above, here we conducted a series of analyses to examine the effect of viewing condition on the (1) HVA, (2) VMA, (3) HVA angular extent, and (4) VMA angular extent:

(1) To test the possible difference between monocular and binocular viewing conditions with respect to the HVA, we conducted a two-way repeated-measures ANOVA (HM vs. VM × 2 viewing conditions) on all three estimates. Figures 10a–c shows SF estimates at the cardinal locations averaged across observers for the monocular and binocular viewing conditions. As in Figure 4, we found a main effect of meridian, indicating a clear HVA for SF threshold (Figure 10a; *F*(1, 6) = 66.33, *p* < 0.001, η^2^_p_ = .92) and SF cutoff (Figure 10b; *F*(1, 6) = 61.54, *p* < 0.001, η^2^_p_ = .91) but not for slope (Figure 10c; *F*(1, 6) < 1). Performance at the HM (LHM and RHM combined) was significantly better than at the VM (UVM and LVM combined) for both SF threshold (HM: 7.33 ± 0.96 cpd; VM: 5.43 ± 0.83 cpd) and SF cutoff (HM: 9.33 ± 0.97 cpd; VM: 6.83 ± 0.81 cpd). Viewing condition had a marginal effect on SF threshold (*F*(1, 6) = 4.44, *p* = 0.080, η^2^_p_ = .43), with higher SF threshold estimates at cardinal locations when tested under binocular (6.51 ± .83 cpd) than under monocular (6.11 ± .92 cpd) viewing condition. We found no significant difference at the cardinal locations between the binocular and monocular viewing conditions in SF cutoff (*F*(1, 6) = 3.14, *p* = 0.127, η^2^_p_ = .34) or in slope (*F*(1, 6) < 1). Importantly, viewing condition did not interact with the effect of location (*F*(1, 6) < 1 for SF threshold, cutoff, and slope), indicating a similar HVA under monocular and binocular viewing conditions.

(2) To test the possible difference between monocular and binocular viewing conditions with respect to the VMA, we conducted a two-way repeated-measures ANOVA (UVM vs. LVM × 2 viewing conditions). As expected, there was a significant VMA for SF thresholds, with significantly higher SF thresholds at the LVM (5.82 ± 0.90 cpd) than at the UVM (5.07 ± 0.85 cpd) (Figure 10a; *F*(1, 6) = 9.23, *p* = 0.023, η^2^_p_ = .61). No significant effect was found for SF cutoff estimates (Figure 10b; *F*(1, 6) = 3.29, *p* = 0.120, η^2^_p_ = .35) or slope (Figure 10c; *F*(1, 6) < 1). When the stimuli were presented on the VM, there was a binocular advantage for SF thresholds (*F*(1, 6) = 9.56, *p* = 0.021, η^2^_p_ = .61; binocular: 5.61 ± 0.84 cpd; monocular: 5.25 ± 0.84 cpd), but not for SF cutoff (*F*(1, 6) = 2.28, *p* = 0.182, η^2^_p_ = .28) or slope (*F*(1, 6) < 1). Importantly, viewing condition did not interact with the effect of location (*F*(1, 6) < 1 for SF threshold, cutoff, and slope), indicating a similar VMA under monocular and binocular viewing conditions.

Figure 10 shows individual HVA ratios (Figures 10d–f; HM divided by VM) and VMA ratios (Figures 10g–i; LVM divided by UVM) as a function of viewing condition. Consistent with Figure 4, all seven observers showed a clear HVA and VMA for SF threshold and SF cutoff estimates (i.e., ratios are higher than 1), with no clear difference in slope (ratios distributed around 1). Importantly, HVA and VMA ratios were distributed along the diagonal line, indicating similar asymmetries under monocular and binocular viewing conditions.

(3) To examine a possible difference between monocular and binocular viewing conditions with respect to the angular extent of the HVA, we used linear mixed-effects models, with angular distance and viewing condition as predictors and participants as a random effect. Figure 11 shows SF threshold (Figure 11a) and SF cutoff (Figure 11b) averaged across lower and upper hemifields as a function of the angular distance from the VM. As in Figures 7a,b, both SF threshold and SF cutoff estimates increased linearly with angular distance (SF threshold—intercept: *t*(94) = 25.61, *p* < 0.001, CI [.6684, .7807]; angular distance: *t*(94) = 7.78, *p* < 0.001, CI [.0011, .0019]; SF cutoff—intercept: *t*(94) = 41.37, *p* < 0.001, CI [.7965, .8768]; angular distance: *t*(94) = 15.44, *p* < 0.001, CI [.0015, .0019]). Moreover, viewing condition had a significant effect on both SF estimates (SF threshold: *t*(94) = 3.01, *p* = 0.003, CI [.0055, .0266]; SF cutoff: *t*(94) = 3.06, *p* = 0.003, CI [.0063, .0295]), which were overall higher in the binocular (SF threshold: 6.35 ± 0.92 cpd; SF cutoff: 8.23 ± 1.06 cpd) than monocular viewing condition (SF threshold: 5.90 ± 1.05 cpd; SF cutoff: 7.89 ± 0.97 cpd). Importantly, viewing condition did not interact with the effect of angular distance for either SF estimates (all *p* values > 0.1 and CIs including 0). Participants’ SF threshold (in log cpd) was equal to .7085 + .00151 * angular distance in the monocular viewing condition and to .7406 + .00150 * angular distance in the binocular viewing condition (Figure 11a). Participants’ SF cutoff (in log cpd) was equal to .8188 + .00186 * angular distance in the monocular viewing condition and to .8545 + .00147 * angular distance in the binocular viewing condition (Figure 11b).

(4) Finally, we assessed whether the angular extent of the VMA (i.e., upper vs. lower visual fields) differed under monocular and binocular viewing conditions. Linear mixed-effects models included angular distance, visual field (upper vs. lower), and viewing conditions (monocular vs. binocular) as predictors and participants as random effect. Figure 11 shows SF threshold (Figure 11c) and SF cutoff (Figure 11d) estimates plotted separately for upper and lower visual field locations as a function of the angular distance from the VM under either monocular or binocular viewing conditions. First, as expected, SF estimates linearly increased as the angular distance from the VM increased for both SF threshold (Figure 11c; intercept: *t*(188) = 25.61, *p* < 0.001, CI [.6688, .7804]; angular distance *t*(188) = 7.78, *p* < 0.001, CI [.0011, .0019]) and SF cutoff (Figure 11d; intercept: *t*(188) = 41.40, *p* < 0.001, CI [.7968, .8765]; angular distance: *t*(188) = 15.58, *p* < 0.001, CI [.0015, .0019]). As in Figures 7c,d, SF estimates were significantly higher in the lower than in the upper VF for both SF estimates (SF threshold: *t*(188) = 3.95, *p* < 0.001, CI [.0176, .0526]; SF cutoff: *t*(188) = 2.91, *p* = 0.004, CI [.0102, .0533]), with the effects of upper-lower VF interacting with angular distance (SF threshold: *t*(188) = 3.82, *p* < 0.001, CI [.0002, .0006]; SF cutoff: *t*(188) = 2.14, *p* = 0.034, CI [.00003, .0006]). All participants showed a steeper linear slope with angular distance in the upper VF than in the lower VF for both SF threshold (Figure 12a) and SF cutoff (Figure 12b) estimates. Finally, viewing condition was associated with a binocular advantage (SF threshold: *t*(188) = 3.14, *p* = 0.002, CI [.0060, .0261]; SF cutoff: *t*(188) = 2.95, *p* = 0.004, CI [.0059, .0298]) but did not interact with either visual field or angular distance (*p* values > 0.1 and CIs including 0). This binocular advantage in acuity was observed in all participants, for both SF threshold (Figure 12c) and SF cutoff (Figure 12d) estimates. Thus, although visual acuity was overall lower under monocular viewing condition, we observed a similar linear decrease in the upper-lower asymmetry (i.e., VMA) with angular distance under binocular and monocular viewing conditions (see Figures 11c,d for the corresponding linear equations).

Taken together, all of these analyses reveal that asymmetries in visual acuity across perifoveal locations (i.e., 10 deg eccentricity) are present regardless of whether participants are tested binocularly or monocularly.

## Discussion

In the present study, we investigated how visual acuity varies with polar angle. To do so, we measured orientation discrimination performance for ±45° oriented, suprathreshold gratings presented at isoeccentric (10 deg eccentricity) locations, every 15° of polar angle. The angular extent of visual asymmetries was characterized as variations in SF thresholds, SF cutoffs, and in the psychometric slope. In the following sections, we summarize and discuss the three main findings revealed by this study.

First, we found clear evidence of HVA and VMA in acuity, with better SF threshold and cutoff estimates at the HM than the VM—the HVA—as well as at the lower VM than the upper VM—the VMA: Observers were more sensitive to stimuli presented at the HM than at the VM and at the LVM than the UVM. These variations in sensory thresholds across cardinal locations are consistent with previous performance fields studies (e.g., Abrams et al., 2012; Altpeter et al., 2000; Baldwin et al., 2012; Cameron et al., 2002; Carrasco et al., 2001, 2002, 2004; Corbett & Carrasco, 2011; Fuller et al., 2008; Fuller & Carrasco, 2009; Greenwood et al., 2017; Himmelberg et al., 2020; Mackeben, 1999; Montaser-Kouhsari & Carrasco, 2009; Nazir, 1992; Petrov & Meleshkevich, 2011; Pointer & Hess, 1989; Rijsdijk et al., 1980; Traquair, 1938; Wallis & Bex, 2012). Moreover, we found that neither the HVA nor the VMA were associated with a consistent change in the slope of the psychometric function. The few studies on performance fields that have also assessed potential differences in psychometric slope across cardinal locations found inconsistent results: on the one hand, consistent with our findings, no difference in psychometric slopes between the upper and lower VM for perceived contrast (Fuller et al., 2008) and, on the other hand, steeper psychometric slopes for the VM than the HM and for the upper than the lower VM in contrast sensitivity (Cameron et al., 2002) but inversely steeper slopes for the HM than the VM and for the lower than the upper VM in illusory motion (Fuller & Carrasco, 2009).

Second, we showed that the angular extent of the HVA and of the VMA decreased gradually as stimuli were moved away from the VM. Thus, a consistent gradual change in visual processing as a function of polar angle not only exists for contrast sensitivity (Abrams et al., 2012; Baldwin et al., 2012) but also for spatial resolution–i.e., for two fundamental visual dimensions mediating performance in many perceptual tasks. This gradual change is consistent with behavioral findings showing similar performance levels at intercardinal (± 45° polar angle) locations (e.g., Abrams et al., 2012; Altpeter et al., 2000; Baldwin et al., 2012; Cameron et al., 2002; Carrasco et al., 2001; Corbett & Carrasco, 2011; Mackeben, 1999; Nazir, 1992; Talgar & Carrasco, 2002), as well as with functional MRI activity (Liu et al., 2006) and cortical magnification (Benson, Kupers, Barbot, Carrasco, & Winawer, 2020) in early visual cortex (i.e., V1/V2). Specifically, surface area in early visual cortex gradually decreases as a function of the angular distance from the HM, reflecting both the HVA and VMA. This gradual change in cortical magnification suggests a tight link between cortical topography and visual perception (Benson et al., 2020).

Third, we found similar performance fields regardless of whether observers were tested monocularly or binocularly. This result is consistent with reported (but unpublished) data from our lab showing no difference in performance fields for contrast sensitivity under binocular and monocular viewing conditions (Carrasco et al., 2001). Thus, we can rule out horizontal disparity as a potential source of performance heterogeneities across the visual field. We observed a binocular advantage consistent with studies reporting binocular enhancement for acuity tasks on the order of 5% to 10% for high-contrast acuity stimuli (e.g., Cagenello, Halpern, & Arditi, 1993; Campbell & Green, 1965; Home, 1978; Pardhan, 2003; Sabesan, Zheleznyak, & Yoon, 2012; Zlatkova, Anderson, & Ennis, 2001). For example, Campbell and Green (1965) reported a binocular advantage in visual acuity of ∼7% when comparing monocular (57 cpd) and binocular (61 cpd) acuity limit. Similarly, we observed a binocular advantage of ∼7% across isoeccentric locations when comparing SF threshold estimates under monocular (5.90 cpd) and binocular (6.35 cpd) viewing conditions. Note that binocular summation can vary substantially in magnitude depending on stimulus and task properties (Baker, Lygo, Meese, & Georgeson, 2018). For example, binocular summation in threshold contrast detection results in a larger binocular advantage than in acuity tasks, typically around √2 (∼40%) (e.g., Campbell & Green, 1965; Home, 1978; Sabesan et al., 2012). Moreover, the binocular advantage in acuity tasks is more pronounced in the periphery than the fovea (Zlatkova et al., 2001) and is reduced as stimulus contrast increases, which suggests that binocular enhancement in acuity can be largely explained by threshold contrast summation (Cagenello et al., 1993; Home, 1978). Importantly, whereas we observed the typical binocular advantage in visual acuity, viewing conditions did not influence the linear change in SF estimates and visual asymmetries with polar angle.

The present findings relate to evidence of performance inhomogeneities in spatial resolution tasks. For instance, the asymmetries in SF processing we observed could account for the finding that the magnitude of the HVA in a Landolt-square acuity task increases as gap size decreases (Carrasco et al., 2002), as it would rely on higher SFs. Similarly, both the HVA and VMA are observed in the detection of small acuity stimuli (De Lestrange-Anginieur & Kee, 2020) and Snellen E letters (Altpeter et al., 2000). The present results also relate to texture segmentation tasks, in which performance is constrained by the spatial resolution of the visual system and the scale of the texture target: Performance peaks at mid-eccentricity, where resolution is optimal for the scale of the texture target, and drops at more peripheral locations, where resolution is too low and at more foveal locations where resolution is too high, known as the central performance drop (CPD; Barbot & Carrasco, 2017; Carrasco, Loula, & Ho, 2006; Carrasco & Barbot, 2014; Gurnsey, Pearson, & Day, 1996; Jigo & Carrasco, 2018; Morikawa, 2000; Poirier & Gurnsey, 2005; Potechin & Gurnsey, 2003; Talgar & Carrasco, 2002; Yeshurun, Montagna, & Carrasco, 2008; Yeshurun & Carrasco, 1998, 2000, 2008). Selectively removing high SFs from the stimulus display eliminates the CPD (Morikawa, 2000). Likewise, selectively adapting to high SFs reduces the CPD and shifts the performance peak toward central locations (Barbot & Carrasco, 2017; Carrasco et al., 2006). Consistent with the asymmetries in SF processing we observed here, texture segmentation performance peaks at farther eccentricities in the lower than the upper VM (Talgar & Carrasco, 2002). Moreover, asymmetries in visual processing are not only present at the encoding stage of visual information, affecting SF discrimination and perceived SF, but also in visual short-term memory (Montaser-Kouhsari & Carrasco, 2009).

The gradual emergence of the HVA and VMA for visual acuity as we move from the HM toward the VM further challenges the idea of a constant upper versus lower visual field asymmetry. Changes in performance across isoeccentric locations have been described as an ellipse (Anderson, Cameron, & Levine, 2014; Engel, 1971; Harvey & Pöppel, 1972; Pöppel & Harvey, 1973; Pretorius & Hanekom, 2006; Wertheim, 1894). Although the horizontal elongation of the elliptical performance field can capture the HVA, an elliptical model cannot capture the robust VMA between upper and lower visual fields observed in the present study, as well as in many other studies (e.g., Abrams et al., 2012; Altpeter et al., 2000; Baldwin et al., 2012; Cameron et al., 2002; Carrasco et al., 2001; Corbett & Carrasco, 2011; Fuller et al., 2008; Himmelberg et al., 2020; Lakha & Humphreys, 2005; Mackeben, 1999; Montaser-Kouhsari & Carrasco, 2009; Nazir, 1992; Pointer & Hess, 1989; Rijsdijk et al., 1980; Talgar & Carrasco, 2002; von Grunau & Dube, 1994). Note that the ellipse model (Anderson et al., 2014) did not take into account important stimulus parameters (e.g., eccentricity, SF, stimulus size, and set size) that can determine whether a VMA is absent or present as well as its magnitude (e.g., Baldwin et al., 2012; Cameron et al., 2002; Carrasco et al., 2001; Himmelberg et al., 2020; Lakha & Humphreys, 2005; Liu et al., 2006; Rijsdijk et al., 1980). Therefore, the ellipse model does not suffice to capture asymmetries around the visual field.

The gradual emergence of the HVA and VMA also highlights the need to reexamine the conclusions of some studies reporting that the VMA reflects an overall upper versus lower visual field asymmetry, regardless of the angular position of the stimulus. Upon inspection, it is clear that such field asymmetries are driven by locations at the VM, as stimuli were only presented exactly at the VM (e.g., Danckert & Goodale, 2001; Edgar & Smith, 1990; Fortenbaugh, Silver, & Robertson, 2015; He et al., 1996; McAnany & Levine, 2007; Rubin, Nakayama, & Shapley, 1996; Schmidtmann, Logan, Kennedy, Gordon, & Loffler, 2015; Thomas & Elias, 2011) or near the VM (e.g., Levine & McAnany, 2005). Visual asymmetries between the upper and lower visual field could, to some degree, reflect ecological constraints. The lower visual field generally contains more visual information than the upper visual field and may be more important for survival. For instance, the sky would take up a significant portion of the upper visual field under most viewing conditions, at least in primates living outside the natural forest (Tootell, Switkes, Silverman, & Hamilton, 1988). It has been proposed that the upper and lower visual fields are functionally specialized for far and near vision, respectively, such that stimuli are processed more efficiently in the lower than in the upper visual field (Previc, 1990). Nevertheless, the present findings, along with those in contrast sensitivity (Abrams et al., 2012; Baldwin et al., 2012), indicate that the asymmetry between the upper and lower visual fields should be described in terms of the polar angular position of visual information.

Differences in visual processing at isoeccentric locations can be as pronounced as differences across eccentricities (Baldwin et al., 2012; Barbot, Abrams, & Carrasco, 2019; Carrasco et al., 2001; Himmelberg et al., 2020; Strasburger, Rentschler, & Juttner, 2011). For instance, contrast sensitivity nearly halves when stimuli are moved from 5 deg to 10 deg along the HM (Virsu & Rovamo, 1979) or when moving stimuli from the HM to the UVM at isoeccentric locations (Abrams et al., 2012). A recent study shows that contrast sensitivity decreases by a third when doubling eccentricity along the HM (4.5 deg vs. 9 deg) or when comparing HM and VM at 4.5 deg eccentricity (Himmelberg et al., 2020). Thus, to eliminate differences in sensory factors when assessing performance in visual tasks, it does not suffice to place stimuli at the same eccentricity. Moreover, the lack of significant differences along the intercardinal (± 45° polar angle) meridians (e.g., Abrams et al., 2012; Altpeter et al., 2000; Benson et al., 2020; Cameron et al., 2002; Carrasco et al., 2001; Corbett & Carrasco, 2011; Liu et al., 2006; Mackeben, 1999; Nazir, 1992; Talgar & Carrasco, 2002) has been used to collapse performance across intercardinal isoeccentric locations (e.g., Barbot & Carrasco, 2017; Guzman-Martinez, Grabowecky, Palafox, & Suzuki, 2011; Liu & Mance, 2011; Montagna, Pestilli, & Carrasco, 2009; Sawaki & Luck, 2013; Yashar, White, Fang, & Carrasco, 2017). It is worth noting that although visual field asymmetries linearly decrease with the angular distance from the vertical meridian and generally become negligible by the intercardinal (± 45° polar angle) meridians, they might still be present and may be worth checking for the specific task at hand. In addition, whereas the overall pattern in visual asymmetries is consistent across participants, the magnitude of visual asymmetries differs among individuals (e.g., Abrams et al., 2012; Baldwin et al., 2012; Carrasco et al., 2001; Himmelberg et al., 2020; Purokayastha et al., 2020; Strasburger et al., 2011; Wertheim, 1894).

Despite similarities in the magnitude of change in visual performance across eccentricity and polar angle, distinct mechanisms might mediate the HVA and the VMA. Whereas increased internal noise can account for the reduction in contrast sensitivity with eccentricity, differences across isoeccentric locations seem to reflect asymmetries in the efficiency of visual filters, particularly for high SFs (Barbot et al., 2019). Moreover, the HVA is present as early as at the retinal receptors (Kupers, Carrasco, & Winawer, 2019), but the VMA only emerges at the midget retinal ganglion cells (Kupers, Benson, Carrasco, & Winawer, 2020). Finally, we observed a lack of correlation between HVA and VMA ratios, consistent with a study that evaluated visual asymmetries in contrast sensitivity (Himmelberg et al., 2020).

What are the physiological substrates underlying performance fields? Starting at the level of the human eye, optical quality is not uniform across the retina (e.g., Curcio, Sloan, Kalina, & Hendrickson, 1990; Jaeken & Artal, 2012; Polans, Jaeken, McNabb, Artal, & Izatt, 2015; Song, Chui, Zhong, Elsner, & Burns, 2011; Thibos, Still, & Bradley, 1996; Zheleznyak, Barbot, Ghosh, & Yoon, 2016). Optical factors degrade retinal image quality, which can result in neural insensitivity to high-SF information (e.g., Barbot et al., 2020; Sabesan, Barbot, & Yoon, 2017; Sabesan & Yoon, 2009; Sawides, de Gracia, Dorronsoro, Webster, & Marcos, 2011). Both defocus and higher-order aberrations increase with eccentricity, with some differences as a function of polar angle (Atchison & Scott, 2002; Lundström, Mira-Agudelo, & Artal, 2009). At the level of the retina, cone density becomes sparser with eccentricity, due to increased size and larger gaps between cones, and decreases by ∼30% between the HM and VM at a fixed eccentricity (Curcio et al., 1990; Song et al., 2011).

A computational observer model has been used to evaluate the extent to which these optical and retinal factors can explain performance differences in contrast sensitivity with polar angle (Kupers et al., 2019). To account for the 30% increase in contrast sensitivity between the UVM and the HM for stimuli (4 cpd) presented at 4.5 deg eccentricity (Cameron et al., 2002), the model required an increase by ∼7 diopters of defocus or a reduction by 500% in cone density, which exceeds by far the variations observed in human eyes. Variations in retinal ganglion cell density also correlate with performance fields, with midget retinal ganglion cells density being 1.4 times greater along the HM than the VM (Curcio & Allen, 1990; Watson, 2014). However, including disparities in these cells still accounts from a small fraction of performance fields (Kupers et al., 2020).

At the level of the lateral geniculate nucleus (Connolly & Van Essen, 1984) and primary visual cortex (V1; Van Essen, Newsome, & Maunsell, 1984; but see Adams & Horton, 2003), there is a greater representation of the area around the HM than the VM. At the cortical level, there is 40% lower BOLD amplitude in V1 for visual stimuli presented on the UVM compared to the LVM (Liu et al., 2006). Consistent with behavioral findings, this asymmetry was observed only for high-SF stimuli, not for low-SF stimuli. Furthermore, performance fields could also reflect differences in the geometry of the visual cortex. For instance, more cortical area is devoted to representing the HM than the VM (Benson et al., 2012; Silva et al., 2018), which could account for the HVA. More cortical area is also devoted to representing the upper versus lower visual field within 1–6 deg eccentricity (Benson et al., 2020), which could account for the VMA. This difference decreases gradually with the angular distance from the VM and is no longer present by the intercardinal (± 45°) meridians, which could account for the present findings. In summary, whereas meridional effects are observed as early as the human eye, these front-end factors can account for only a small fraction of performance fields, which are likely due to asymmetries in visual processing across polar angle being amplified at cortical processing stages (Benson et al., 2020). Computational models are needed to quantify the degree to which these cortical factors account for many psychophysical findings of visual field asymmetries.

Our findings reveal that asymmetries in visual acuity emerge gradually with angular distance at isoeccentric, perifoveal (i.e., 10 deg eccentricity) locations. Although we did not test other eccentricities, performance fields have been reported over a wide range of eccentricities, from ∼2 deg to 60 deg (e.g., Abrams et al., 2012; Altpeter et al., 2000; Baldwin et al., 2012; Cameron et al., 2002; Carrasco et al., 2001, 2002; Corbett & Carrasco, 2011; Fuller et al., 2008; Himmelberg et al., 2020; Mackeben, 1999; Montaser-Kouhsari & Carrasco, 2009; Nazir, 1992; Pointer & Hess, 1989; Rijsdijk et al., 1980; Strasburger et al., 2011; Talgar & Carrasco, 2002; see also Table 1 in Baldwin et al., 2012). The magnitudes of both the HVA and the VMA vary with eccentricity. For instance, circular isoperformance lines are usually observed near the fovea and turn into horizontally elongated fields as stimuli are moved to the periphery (e.g., Baldwin et al., 2012; Harvey & Pöppel, 1972; Pointer & Hess, 1989; Pöppel & Harvey, 1973; Strasburger et al., 2011). Baldwin et al. (2012) measured contrast sensitivity as a function of eccentricity, SF, and polar angle and found that the decline in contrast sensitivity with eccentricity is bilinear within the central visual field. Specifically, they found that the attenuation in sensitivity as a function of eccentricity (0–4.5 deg) and polar angle (45° steps) had the form of a witch's hat, with a steep initial decline near the fovea followed by a shallower decline in sensitivity. This bilinear decline in sensitivity with eccentricity was steeper along the upper VM, with the slope of the lower VM being almost half that for the upper VM for the shallower part of the witch's hat. This finding is consistent with the VMA becoming more pronounced with eccentricity.

Measuring the conspicuity of visual stimuli across eccentricity as well as at isoeccentric locations provides a framework for how the visual system processes information across the visual field. Characterizing how visual performance varies at eccentric and isoeccentric locations has profound implications not only for our understanding of visual perception but also for ergonomic and human factors applications. For instance, when designing devices for drivers, pilots, radiologists, air traffic controllers, and many others, one should take into account perceptual asymmetries across the visual field and tailor displays for optimal speed and accuracy. Although visual performance measures, such as acuity and contrast sensitivity, are only marginally correlated (Poggel, Treutwein, Calmanti, & Strasburger, 2012a, 2012b), and the magnitude of visual field asymmetries varies with stimulus SF, stimulus eccentricity, set size (e.g., Baldwin et al., 2012; Cameron et al., 2002; Carrasco et al., 2001; Himmelberg et al., 2020), and across individuals (e.g., Abrams et al., 2012; Baldwin et al., 2012; Carrasco et al., 2001; Himmelberg et al., 2020; Purokayastha et al., 2020; Strasburger et al., 2011; Wertheim, 1894), it would be preferable to present critical information along the horizontal meridian rather than near the upper VM, given that it corresponds to the region of the visual field with the poorest contrast sensitivity and spatial resolution. Future studies are needed to fully characterize visual performance fields across different eccentricities, polar angle, and tasks.

## Conclusion

Aiming to understand how limits in visual processing change around our visual field, we measured orientation discrimination performance of high-contrast gratings varying in SF at 24 isoeccentric and equidistant peripheral locations. The present results reveal that both the HVA and VMA in visual acuity are most pronounced at the vertical meridian and decrease gradually approaching the horizontal meridian. Furthermore, this pattern is the same for both monocular and binocular viewing, hence ruling out differences in horizontal disparity as a possible source of these performance fields. These results provide a more complete picture regarding how spatial resolution differs across our visual field, a fundamental dimension constraining visual performance in many tasks. These location-dependent asymmetries in visual acuity, as well as those in contrast sensitivity (e.g., Abrams et al., 2012; Baldwin et al., 2012), have important perceptual consequences that should be accounted for in current models of visual perception (e.g., Akbas & Eckstein, 2017; Bradley, Abrams, & Geisler, 2014; Kupers et al., 2019, 2020; Schira, Tyler, Spehar, & Breakspear, 2010; Schutt & Wichmann, 2017).
